# *GRIN1* variants associated with neurodevelopmental disorders reveal channel gating pathomechanisms

**DOI:** 10.1111/epi.17776

**Published:** 2023-10-17

**Authors:** Lotten Ragnarsson, Zihan Zhang, Sooraj S. Das, Poanna Tran, Åsa Andersson, Vincent des Portes, Cecilia Desmettre Altuzarra, Ganaelle Remerand, Audrey Labalme, Nicolas Chatron, Damien Sanlaville, Gaetan Lesca, Victor Anggono, Irina Vetter, Angelo Keramidas

**Affiliations:** ^1^ Institute for Molecular Bioscience University of Queensland St. Lucia Queensland Australia; ^2^ Clem Jones Centre for Ageing Dementia Research, Queensland Brain Institute University of Queensland Brisbane Queensland Australia; ^3^ Neuropaediatrics Department Femme Mère Enfant Hospital Lyon France; ^4^ Department of Pediatrics Centre Hospitalier Universitaire (CHU) Besançon France; ^5^ Department of Neonatology, Estaing Hospital Centre Hospitalier Universitaire (CHU) Clermont‐Ferrand France; ^6^ Service de Génétique, Hospices Civils de Lyon Groupement Hospitalier Est Bron France

**Keywords:** developmental delay, epilepsy, NMDA receptors

## Abstract

**Objective:**

*N*‐methyl‐d‐aspartate (NMDA) receptors are expressed at synaptic sites, where they mediate fast excitatory neurotransmission. NMDA receptors are critical to brain development and cognitive function. Natural variants to the *GRIN1* gene, which encodes the obligatory GluN1 subunit of the NMDA receptor, are associated with severe neurological disorders that include epilepsy, intellectual disability, and developmental delay. Here, we investigated the pathogenicity of three missense variants to the *GRIN1* gene, p. Ile148Val (GluN1‐3b[I481V]), p.Ala666Ser (GluN1‐3b[A666S]), and p.Tyr668His (GluN1‐3b[Y668H]).

**Methods:**

Wild‐type and variant‐containing NMDA receptors were expressed in HEK293 cells and primary hippocampal neurons. Patch‐clamp electrophysiology and pharmacology were used to profile the functional properties of the receptors. Receptor surface expression was evaluated using fluorescently tagged receptors and microscopy.

**Results:**

Our data demonstrate that the GluN1(I481V) variant is inhibited by the open pore blockers ketamine and memantine with reduce potency but otherwise has little effect on receptor function. By contrast, the other two variants exhibit gain‐of‐function molecular phenotypes. Glycine sensitivity was enhanced in receptors containing the GluN1(A666S) variant and the potency of pore block by memantine and ketamine was reduced, whereas that for MK‐801 was increased. The most pronounced functional deficits, however, were found in receptors containing the GluN1(Y668H) variant. GluN1(Y668H)/2A receptors showed impaired surface expression, were more sensitive to glycine and glutamate by an order of magnitude, and exhibited impaired block by extracellular magnesium ions, memantine, ketamine, and MK‐801. These variant receptors were also activated by either glutamate or glycine alone. Single‐receptor recordings revealed that this receptor variant opened to several conductance levels and activated more frequently than wild‐type GluN1/2A receptors.

**Significance:**

Our study reveals a critical functional locus of the receptor (GluN1[Y668]) that couples receptor gating to ion channel conductance, which when mutated may be associated with neurological disorder.

**Key Points**

- Variants that give rise to epilepsy and neurodevelopmental delay were identified in the *GRIN1* gene that encodes the GluN1 subunit of the NMDA receptor
- All three variants showed altered block by ketamine, memantine, and MK‐801
- The variants p.Ala666Ser and p.Tyr668His increased the sensitivity to the neurotransmitter glycine
- The p.Tyr668His variant reduced surface expression, was also more sensitive to glutamate, and could be activated by either glutamate or glycine alone

## 1 INTRODUCTION

**FIGURE 1:**
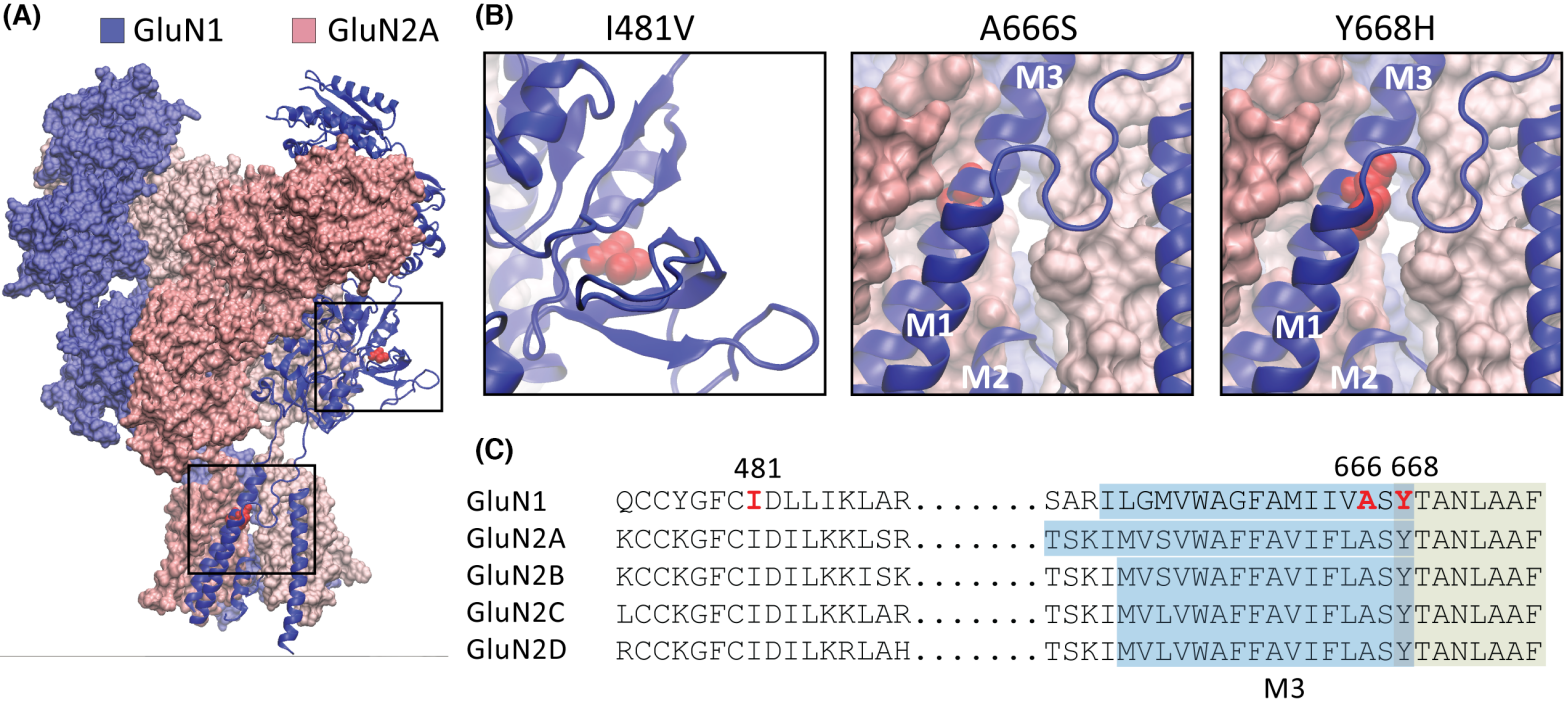
GluN1 missense variants. (A) Schematic representation of the GluN1/2A *N*‐methyl‐d‐aspartate receptor in the agonist‐bound state showing the overall subunit arrangement and the position of the three GluN1 variants. (B) Expanded views of the boxed areas in A showing the amino acid substitutions as red spheres. The schematics were made in Visual Molecular Dynamics software using the 7EOS PDB file.^1^ (C) Segments of amino acid sequences that encompass the three variants showing the sequence identity between the GluN1 and GluN2 subunits. The substituted positions in the GluN1‐3b subunit are shown in red. The M3 transmembrane domain is highlighted in blue, and the contiguous segment that is a key signal transduction element is highlighted in green.

*N*‐methyl‐d‐aspartate (NMDA) receptors are tetrameric assemblies of two obligatory GluN1 subunits and two GluN2 (A–D) or GluN3 (A or B) subunits (Figure 1A). They are located at synaptic sites throughout the brain and spinal cord, where they mediate excitatory neurotransmission.^2^, ^3^ The subunits assemble to form a central cation‐selective ion channel that exhibits relatively high permeability to Ca^2+^ ions.^4^ The channels also exhibit prominent voltage‐dependent channel block by extracellular Mg^2+^ ions, which is relieved by membrane depolarization.^4^, ^5^ Activation of NMDA receptors requires the binding of two neurotransmitter agonists, glutamate and glycine (or d‐serine).^2^, ^3^ Glutamate binds to the GluN2 subunits, whereas glycine binds to the GluN1 and GluN3 subunits.^2^, ^3^, ^6^ Each subunit comprises an amino‐terminal domain (ATD), a ligand‐binding domain (LBD), a transmembrane domain (TMD), and a carboxy‐terminal domain. The TMDs consist of four α‐helical segments; M1, M3, and M4 span the cell membrane, whereas the M2 segment forms a re‐entrant loop. The M3 lines the wall of the ion channel pore, and the M2 segment forms the ion selectivity filter (Figure 1B).^7^

Genetic variants that encode NMDA receptor subunits give rise to diverse neurodevelopmental disorders such as autism spectrum disorder, epilepsy, developmental delay, and intellectual disability.^8^, ^9^ More than 350 variants of the GluN1 subunit have been identified.^10^ The variants mostly occur within the ATDs, LBDs, and TMDs^8^, ^11^, ^12^ and can result in either gain‐ or loss‐of‐function molecular phenotypes.^10^, ^11^, ^12^ Here, we report three newly identified variants of the GluN1 subunit, which we expressed on the GluN1‐3b splice isoform for functional analysis. The missense p. Ile148Val variant is absent from any databases and occurs within the LBD of the receptor. The other two missense, de novo variants, p.Tyr668His and p.Ala666Ser, are located at the boundary between the M3 transmembrane domain and a highly conserved stretch of residues (ASYTANLAAF) that is a key signal transduction element in receptor gating (Figure 1C).^13^, ^14^ Missense variants to the A666 and Y668 positions have been reported previously in other individuals,^12^, ^15^ suggesting that this region of the GluN1 subunit is prone to disease‐causing mutations.

Our functional analysis shows that the GluN1(I481V) variant has minimal effects on receptor function, whereas the two pore variants exhibit gain‐of‐function molecular phenotypes. The GluN1(A666S)/2A receptors show an increased sensitivity to glycine, and impaired channel block by memantine and ketamine, but enhanced block by MK‐801. The GluN1(Y668H)/2A receptors show a decrease in surface expression, an increase in sensitivity to glutamate and glycine, and impaired block by memantine, ketamine, MK‐801, and extracellular Mg^2+^ ions. Remarkably, this variant can be activated by either glycine or glutamate alone. Additionally, this variant exhibited frequent but briefer single‐channel activations, which would predict longer synaptic decay times. Our data show that the GluN1(Y668) position is a structural hub that couples receptor gating to ionic flux and regulates open channel block by Mg^2+^ and clinically relevant drugs.

## 2 MATERIALS AND METHODS

### 2.1 HEK293 cell culture and transfection

Human GluN1 (isoform GluN1‐3b) and GluN2A wild‐type (WT) cDNAs (pRK5 expression vector) were used for electrophysiological analysis. The plasmid encoding superecliptic pHluorin (SEP)‐GluN1‐3b was generated by subcloning rat GluN1‐3b cDNA into the pRK5 vector and subsequently inserting SEP cDNA after the signal peptide. HEK293AD cells were transfected using calcium phosphate coprecipitation or Lipofectamine 2000 with cDNAs encoding the GluN1‐3b, or GluN1‐3b variants along with the GluN2A subunit at a ratio of 1:1. Standard whole‐cell and single‐channel patch‐clamp recordings were carried out in Mg^2+^‐free extracellular solution.

### 2.2 Electrophysiology

Conventional patch‐clamp experiments were carried out at a clamped potential of −70 mV (unless otherwise indicated) in standard whole‐cell and outside‐out patch configurations, whereas automated electrophysiology was done in the whole‐cell configuration at −100 mV.

The Mg^2+^‐free extracellular solution comprised (in mmol·L^−1^): 140 NaCl, 5 KCl, 2 CaCl_2_, .02 Na_2_EDTA, 10 hydroxyethylpiperazine ethane sulfonic acid (HEPES), and 10 d‐glucose. The Mg^2+^‐containing extracellular solution comprised of (in mmol·L^−1^): 140 NaCl, 5 KCl, 2 CaCl_2_, 1 MgCl_2_, 10 HEPES, and 10 d‐glucose. NaOH was used to adjust the extracellular solutions to a pH of 7.4. The intracellular solution for conventional patch‐clamp experiments was composed of (in mmol·L^−1^): 145 CsCl, 2 CaCl_2_, 2 MgCl_2_, 10 HEPES, and 10 ethyleneglycoltetraacetic acid (EGTA), adjusted to pH 7.4 with CsOH. The intracellular solution for automated patch clamp experiments consisted of (in mmol·L^−1^) 140 CsF, 1 EGTA, 5 CsOH, 10 HEPES, and 10 NaCl, pH to 7.3 with CsOH (adjusted to 320 mOsm/L with sucrose).

Single‐receptor and conventional whole‐cell experiments were recorded using an EPC 10 USB HEKA Patch Clamp Amplifier (HEKA, Elekronik), filtered (−3 dB, 4‐pole Bessel) at 5 kHz and sampled at 50 kHz. Recording electrodes were made from borosilicate glass capillaries and heat‐polished to a final resistance of 3–6 MΩ (whole‐cell) or 6–12 MΩ (excised patch) when filled with intracellular solution. The electrodes used for single‐receptor recordings were also coated with a silicone elastomer. Automated whole‐cell patch‐clamp recordings were performed with a QPatch‐16 automated electrophysiology platform (Sophion Bioscience) using single‐hole (QPlate 16 with a standard resistance of 2 ± .4 MΩ).

QUB software was used to analyze single‐channel currents, which were idealized using a resolution dead‐time of 70 μs; the critical shut period that separated one discrete activation from the next was 1000 ms.

Peak current responses to the addition of agonists and antagonists (pore blockers; *I*) were normalized to buffer control (*I*_0_). Half‐maximal effective concentration (EC_50_) and half‐maximal inhibitory concentration (IC_50_) values were determined by plotting difference in peak current (*I*/*I*_0_) against the logarithm of agonist or antagonist concentrations, respectively. Concentration–response data were plotted and fitted to four‐parameter Hill equations using GraphPad Prism software. The reversal potential values that were used to calculate single‐channel conductance were corrected for liquid junction potentials.

Electrospray ionization mass spectrometry was performed on an API 2000 LC/MS/MS system (AB SCIEX) running .1% (vol/vol) formic acid/80% (vol/vol) acetonitrile/H_2_O.

### 2.3 Neuronal culture, transfection, and surface staining

Primary hippocampal neurons were prepared from Sprague Dawley rat embryos at Embryonic Day 18 according to an established protocol.^16^ Every procedure involving animal use adhered to the Australian Code of Practice for the Care and Use of Animals for Scientific Purposes and was approved by the University of Queensland Animal Ethics Committee (2021/AE000511). Cells were dissociated by incubating hippocampi with 30 U of papain suspension at 37°C for 20 min, followed by mechanical trituration with fire‐polished glass Pasteur pipettes. Single‐cell suspensions were plated at a density of 8 × 10^4^ cells on a poly‐l‐lysine‐coated coverslip in Neurobasal growth medium containing 2 mmol·L^−1^ Glutamax, 1% penicillin/streptomycin, and 2% B‐27 supplement. Neurons were maintained in Neurobasal medium inside a humidified 5% CO_2_ tissue culture incubator at 37°C and fed twice per week. Neurons were transfected at 14 days in vitro (DIV) with Lipofectamine 2000 (Invitrogen) and processed at 17 DIV.

To measure the steady‐state expression of NMDA receptors on the plasma membrane, live neurons expressing SEP‐GluN1 were incubated with rabbit anti‐green fluorescent protein (GFP) antibodies (JH4030, 1:250) at 4°C for 30 min.^17^ After washing, neurons were fixed with ice‐cold Parafix (4% paraformaldehyde, 4% sucrose in phosphate‐buffered saline) for 10 min. They were then permeabilized with .25% Triton X‐100, blocked with 10% normal goat serum, and incubated with chicken anti‐GFP antibodies to label total SEP‐GluN1 expression. The surface and total SEP‐GluN1 were visualized by Alexa‐568‐conjugated antirabbit and Alexa‐488‐conjugated antichicken secondary antibodies, respectively. Images were collected with a 63× oil‐immersion objective on a Zeiss LSM510 confocal microscope using ImageJ software.

## 3 RESULTS

### 3.1 Clinical presentation of patients

Clinical features and medical histories of the three patients are available in the supporting text, and example electroencephalograms are shown in Figure S1. The variants were identified through gene panel sequencing.

### 3.2 Cell surface expression levels for WT and GluN1 variants

**FIGURE 2:**
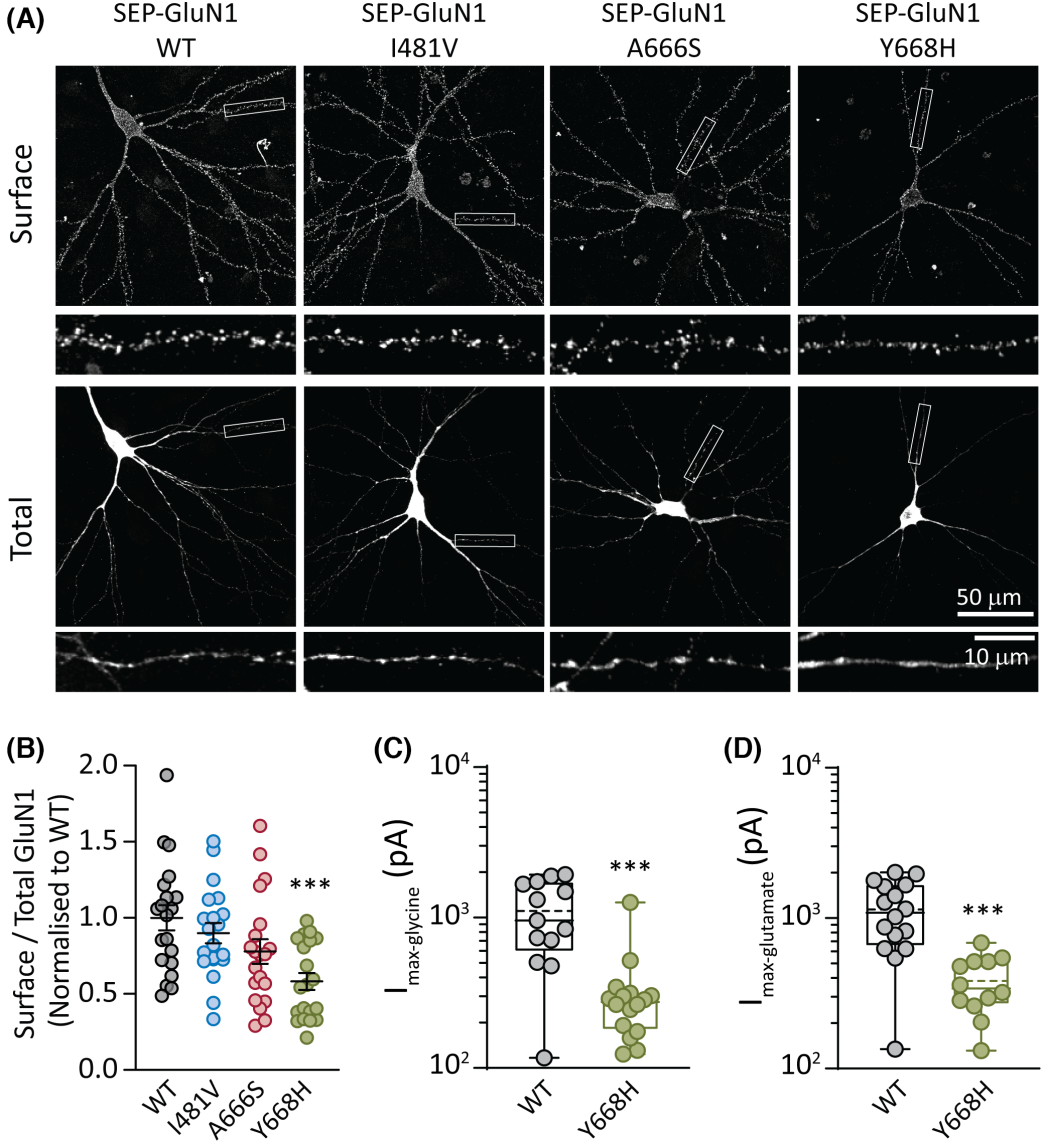
GluN1 variant surface expression. (A) Rat hippocampal neurons transfected with plasmids encoding superecliptic pHluorin (SEP)‐GluN1 (wild‐type [WT]) and the three variant subunits SEP‐GluN1(I481V), SEP‐GluN1(A666S), and SEP‐GluN1(Y668H). Representative images are shown of the surface and total SEP‐GluN1 in a neuron from each group, together with expanded views of the boxed regions, shown below. (B) Quantification of surface expression of the surface/total GluN1 ratio normalized to the value of control neurons expressing SEP‐GluN1 WT. Data are presented as mean ± SEM (WT, *n* = 20 neurons; I481V, *n* = 20; A666S, *n* = 20; Y668H, *n* = 20; from three independent cultures). (C) Quantification of peak whole‐cell currents mediated by the indicated receptors in response to saturating concentrations of glycine (and half‐maximal effective concentration [EC_50_] of glutamate). (D) Quantification of peak whole‐cell currents in response to saturating concentrations of glutamate (and an EC_50_ concentration of glycine). ***p < .001.

Cell surface expression of GluN1 subunits was examined in primary rat hippocampal neurons transfected with cDNAs encoding superecliptic pH‐sensitive GFP (SEP)‐tagged rat GluN1 subunit, either WT, I481V, A666, or Y668H variants. Surface receptors were labeled with anti‐GFP antibodies under nonpermeabilized conditions.^16^ The surface expression of SEP‐GluN1 in neuronal dendrites that expressed GluN1(I481V) and GluN1(A666S) variants was unaffected relative to WT. The surface‐to‐total ratio of WT SEP‐GluN1 was 1.0 ± .08 compared to .90 ± .07 for the GluN1(I481V) (*n* = 20, *p* = .76) and .78 ± .08 (*n* = 20, *p* = .14) for the GluN1(A666S) (Figure 2A,B). By contrast, a significant decrease in receptor surface expression was observed for the GluN1(Y668H) variant (.58 ± .06, *n* = 20, one‐way analysis of variance [ANOVA], *p* < .001; Figure 2A,B).

To assess whether the reduction in surface expression corresponded to a decrease in peak current in the GluN1(Y668H)/2A receptors, we transfected human WT GluN1 or variant GluN1(Y668H) with WT GluN2A subunits in HEK293AD cells and performed whole‐cell patch‐clamp electrophysiology. Whole‐cell currents were elicited by applying a maximal (saturating) concentration of glycine or glutamate along with an ~EC_50_ concentration of the coagonists. The mean peak glycine‐gated current for GluN1/2A WT receptors was 1143 ± 558 pA (*n* = 16 cells) and for GluN1(Y668H)/2A variant receptors was 383 ± 163 pA (*n* = 12 cells, unpaired *t*‐test, *p* < .001; Figure 2C). A similar analysis for glutamate‐gated currents produced a mean peak for WT receptors of 1105 ± 600 pA (*n* = 13 cells) and for GluN1(Y668H)/2A receptor of 323 ± 268 pA (*n* = 16 cells, unpaired *t*‐test, *p* < .001; Figure 2D). These data show a clear correlation between the reduced surface expression level and reduced maximal currents mediated by the GluN1(Y668H)/2A receptors compared to WT receptors.

### 3.3 Glycine sensitivity at WT and GluN1 variants

**FIGURE 3:**
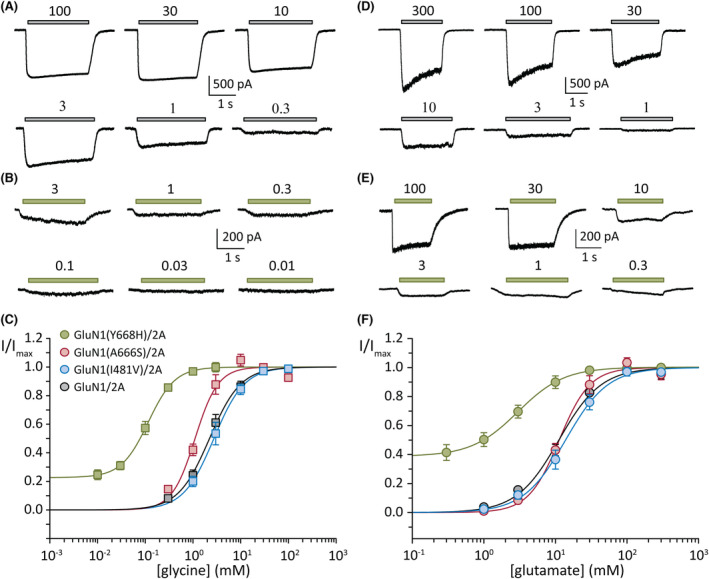
Concentration–response plots of glycine and glutamate. (A, B) Representative whole‐cell currents in response to the indicated micromolar concentrations of glycine (and half‐maximal effective concentration [EC_50_] of glutamate) from cells expressing the wild‐type (WT) GluN1/2A (A) and GluN1(Y668H)/2A variant (B) receptors. (C) Normalized glycine concentration–response plots of group data for the indicated WT and variant receptors. (D, E) Representative whole‐cell currents in response to the indicated micromolar concentrations of glutamate (and an EC_50_ concentration of glycine) from cells expressing the WT GluN1/2A (D) and GluN1(Y668H)/2A variant (E) receptors. (C) Normalized glutamate concentration–response plots of group data for the indicated WT and variant receptors. Currents were obtained at a holding potential of −70 mV.

The sensitivity of the receptors to glycine was tested in transfected HEK293AD cells expressing WT or variant‐containing receptors. Whole‐cell currents were elicited by direct application of a range of glycine concentrations and a constant glutamate concentration of 20 μmol·L^−1^ (WT) or 5 μmol·L^−1^ (GluN1 variants), over each recorded cell, as exemplified for WT GluN1/2A and GluN1(Y668H)/2A receptors (Figure 3A,B). Normalized peak currents were then plotted and fitted to a Hill equation for each cell expressing either WT GluN1/2A, GluN1(I481V)/2A, GluN1(A666S)/2A, or GluN1(Y668H)/2A receptors to obtain the concentration that elicits the half‐maximal current (EC_50_) and the Hill coefficient (*n*_H_; slope at EC_50_; Figure 3C). The WT receptors exhibited a glycine pEC_50_ (negative logarithm of EC_50_) of −.42 ± .26 μmol·L^−1^ (EC_50_ of 3.2 ± 2.2 μmol·L^−1^, *n* = 14) and an *n*_H_ of 1.7 ± .5. Similar parameters were obtained for the GluN1(I481V)/2A receptors, with a glycine pEC_50_ of −.44 ± .25 μmol·L^−1^ (EC_50_ of 3.2 ± 1.9 μmol·L^−1^, *n* = 9, ANOVA, *p* = .797) and an *n*_H_ of 1.7 ± .5. The GluN1(A666S)/2A receptors produced a pEC_50_ of −.08 ± .13 μmol·L^−1^ (EC_50_ of 1.2 ± .4 μmol·L^−1^, *n* = 5, ANOVA, *p* = .018) and an *n*_H_ of 1.5 ± .3, whereas the GluN1(Y668H)/2A variant produced a pEC_50_ of .92 ± .15 μmol·L^−1^ (EC_50_ of .13 ± .04 μmol·L^−1^, *n* = 9, ANOVA, *p* < .001).

We made the notable observation that as glycine concentrations diminished to zero, the response remained at ~23% of the maximum for the GluN1(Y668H)/2A variant receptors, whereas for the other receptors the response diminished to zero (Figure 3C).

### 3.4 Glutamate sensitivity at WT and GluN1 variants

Similar experiments and analyses were conducted to determine the glutamate concentration–response relationship at WT and variant receptors. Whole‐cell currents were recorded over a range of glutamate concentrations, and a constant glycine concentration of 4 μmol·L^−1^ (WT) or 10 μmol·L^−1^ (GluN1 variants), over each recorded cell, as shown for WT GluN1/2A and GluN1(Y668H)/2A receptors (Figure 3D,E). The GluN1/2A receptors produced a glutamate pEC_50_ of −1.2 ± .2 (EC_50_ of 19.6 ± 10.1 μmol·L^−1^, *n* = 13) and an *n*_H_ of 1.4 ± .5. The pEC_50_ and *n*_H_ values for the GluN1(I481V)/2A variant were −1.2 ± .2 (EC_50_ of 20.2 ± 9.8, *n* = 9, ANOVA, *p* = .974) and 1.8 ± .9, and those for the GluN1(A666S)/2A were −1.1 ± .1 (EC_50_ of 13.9 ± 4.6, *n* = 5, ANOVA, *p* = .846) and 2.1 ± .5.

As was observed for glycine sensitivity, the GluN1(Y668H)/2A showed an enhanced sensitivity to glutamate, with a pEC_50_ of −.33 ± .42 (EC_50_ of 3.3 ± 3.2 μmol·L^−1^, *n* = 13, ANOVA, *p* < .001) and an *n*_H_ of 1.2 ± .4. Here too, the current mediated by the GluN1(Y668H)/2A variant remained at ~39% of maximum, unlike that of the other receptors, which diminished to zero with decreasing glutamate concentrations (Figure 3F).

### 3.5 GluN1(Y668H)/2A receptor responds to either glycine or glutamate, without added coagonist

**FIGURE 4:**
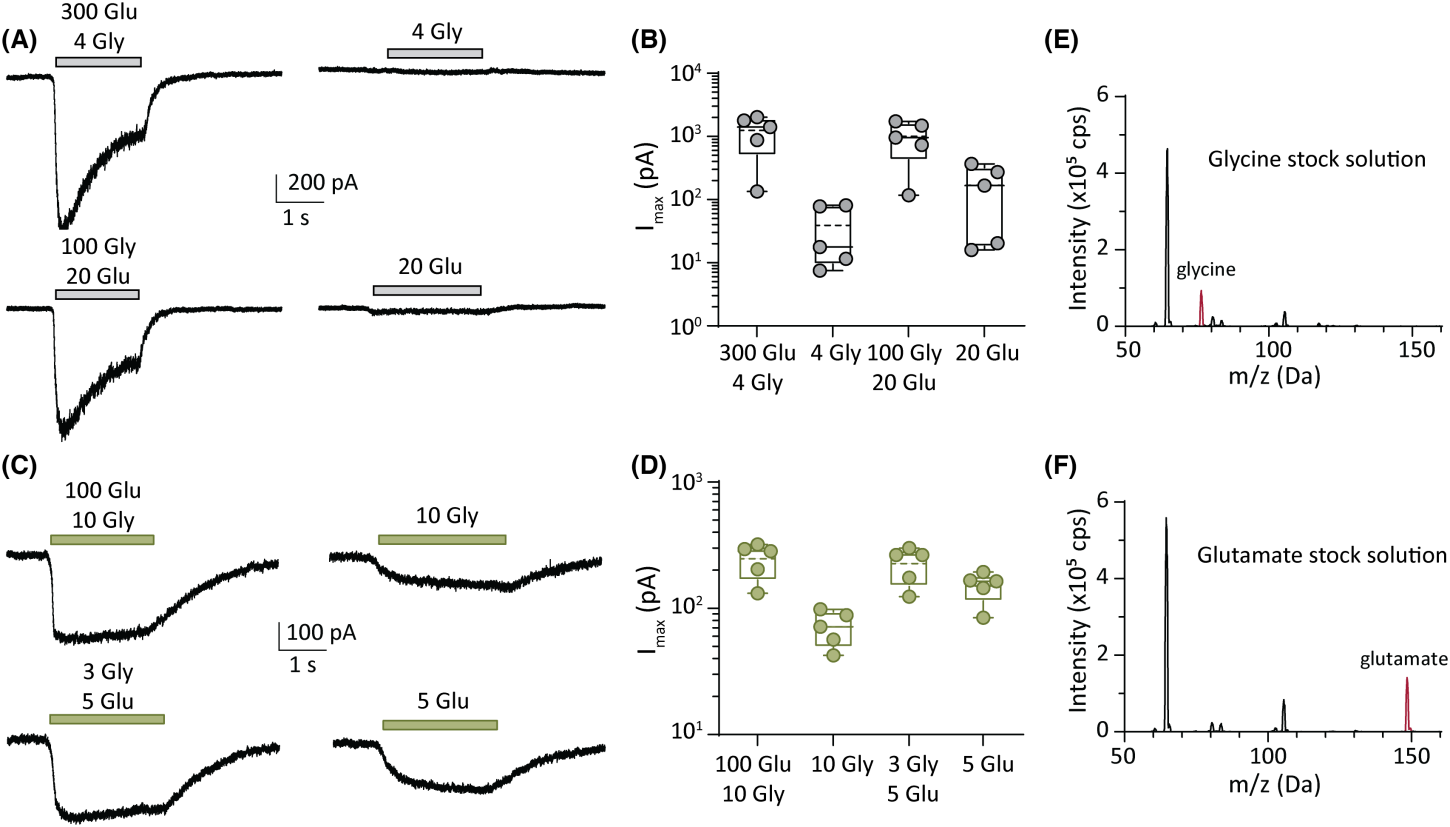
Activation of GluN1(Y668H)/2A by single neurotransmitter. (A) Representative whole‐cell currents in response to the indicated micromolar concentrations of agonists (left) and single agonist (right) for wild‐type (WT) GluN1/2A receptors. (B) Quantification of peak currents from group data showing peak currents from agonist combinations compared to single agonists for WT receptors. (C) Representative whole‐cell currents in response to the indicated micromolar concentrations of agonists (left) and a single agonist (right) for GluN1(Y668H)/2A variant receptors. (D) Quantification of peak currents from group data showing peak currents from agonist combinations compared to single agonists for GluN1(Y668H)/2A variant receptors. (E, F). Mass spectra of the agonist stock solutions used in this study. The glycine stock (E) shows a clear peak at the glycine molecular mass of 75.07 g mol^−1^ (red peak), whereas the glutamate stock (F) shows a peak at the molecular mass of glutamate of 147.13 g mol^−1^ (red peak). Note that the plots in E and F show additional peaks that correspond to other masses in the extracellular solution.

We conducted whole‐cell recordings to determine the origin of the residual current mediated by GluN1(Y668H)/2A receptors at diminishing glycine or glutamate concentrations. For these experiments, currents were recorded as pairs from the same cell. These consisted of a current elicited by the agonist combinations that produced a maximal response, followed by a current elicited by the single agonist that was held constant throughout the concentration–response experiment. At WT receptors, 300 μmol·L^−1^ glutamate plus 4 μmol·L^−1^ glycine produced a mean current of 1243 ± 756 pA (*n* = 5), whereas 4 μmol·L^−1^ glycine alone elicited a mean current of 39 ± 37 pA, representing only ~3% of the maximum. Similarly, 100 μmol·L^−1^ glycine plus 20 μmol·L^−1^ glutamate elicited a mean current of 1007 ± 639 pA (*n* = 5) and 20 μmol·L^−1^ glutamate alone elicited a mean current of 168 ± 154 pA, representing only ~17% of the maximum (Figure 4A,B). These data are in line with the current mediated by WT receptors diminishing toward zero with decreasing agonist concentrations.

Similar experiments were done on the GluN1(Y668H)/2A variant receptors. One hundred micromolars glutamate plus 10 μmol·L^−1^ glycine produced a mean maximal current of 247 ± 78 pA (*n* = 5), and 10 μmol·L^−1^ glycine alone elicited a mean current of 71 ± 23 pA, which was ~29% of the maximum. Three micromolars glycine plus 5 μmol·L^−1^ glutamate produced a maximum current of 226 ± 74 pA (*n* = 5), and 5 μmol·L^−1^ glutamate alone produced a mean current of 151 ± 41 pA, which was ~67% of the maximum (Figure 4C,D). The current that was elicited by single agonists was substantially larger relative to that elicited by both agonists for this variant receptor when compared to the WT receptor. Our data suggest that the persistent currents observed in the concentration–response experiments either might be due to low‐level contamination of coagonist in our stock solutions of glycine and glutamate, as has been suggested for the glycine (but not glutamate) concentration–response relationship at GluN1‐4a(N650K)/2A variant receptors,^18^ or, more remarkably, indicate that the GluN1(Y668H)/2A variant receptors can be activated by either agonist alone.

To determine whether our stock solutions of glutamate and glycine were contaminated by the coagonist, we carried out mass spectrometry on both stock solutions. The spectral plots for both solutions are shown in Figure 4. The glycine stock solution showed a clear peak at the molecular mass of glycine (75.07 g mol^−1^; Figure 4E) but no evidence for the presence of glutamate. Similarly, the mass spectrum of our glutamate stock showed a prominent peak at the molecular mass of glutamate (147.13 g mol^−1^; Figure 4F) and no peak for glycine. These data indicate that contamination by coagonist was not detected in our stock solutions, which supports the interpretation that a single agonist is capable of gating the GluN1(Y668H)/2A variant receptors.

### 3.6 Single‐receptor current properties of WT, GluN1(A666S)/2A, and GluN1(Y668H)/2A receptors

**FIGURE 5:**
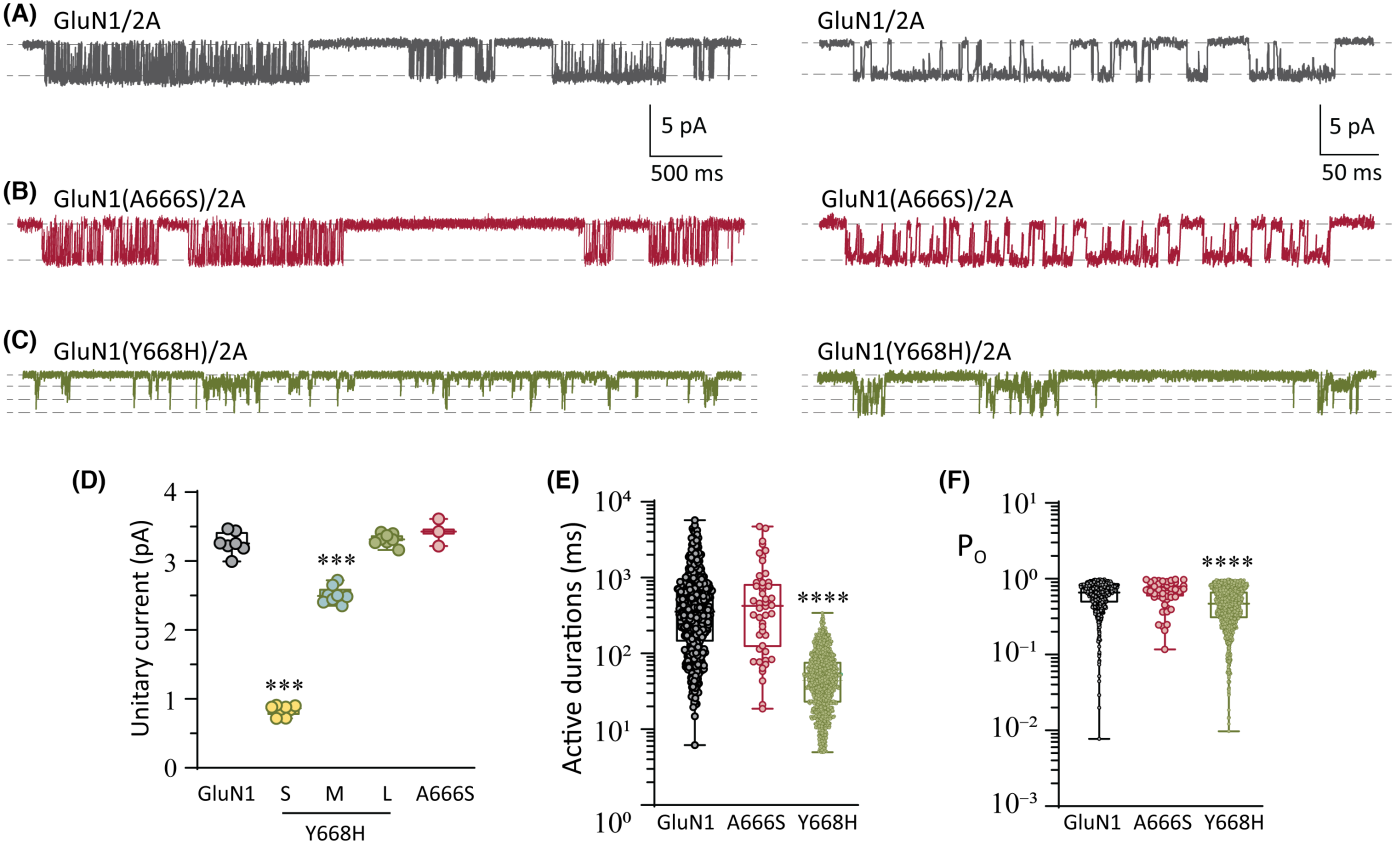
Single‐receptor currents. (A–C) Representative single‐receptor currents recorded from excised outside‐out membrane patches expressing the WT GluN1/2A (A), GluN1(A666S)/2A (B), and GluN1(Y668H)/2A (C). Expanded views of single‐receptor activity are shown on the left. (D) Quantification of unitary current for the indicated receptors. Note that the GluN1(Y668H)/2A variant receptors have a wild‐type‐like amplitude (large [L]) and two smaller amplitudes (small [S] and medium [M]). (E) Plots of discrete activations (active periods) of single‐receptor current for the indicated receptors. (F) Plots of the open state occupancy (P_O_) within active periods for the indicated receptors. ***p < .001, ****p < .0001.

**FIGURE 6:**
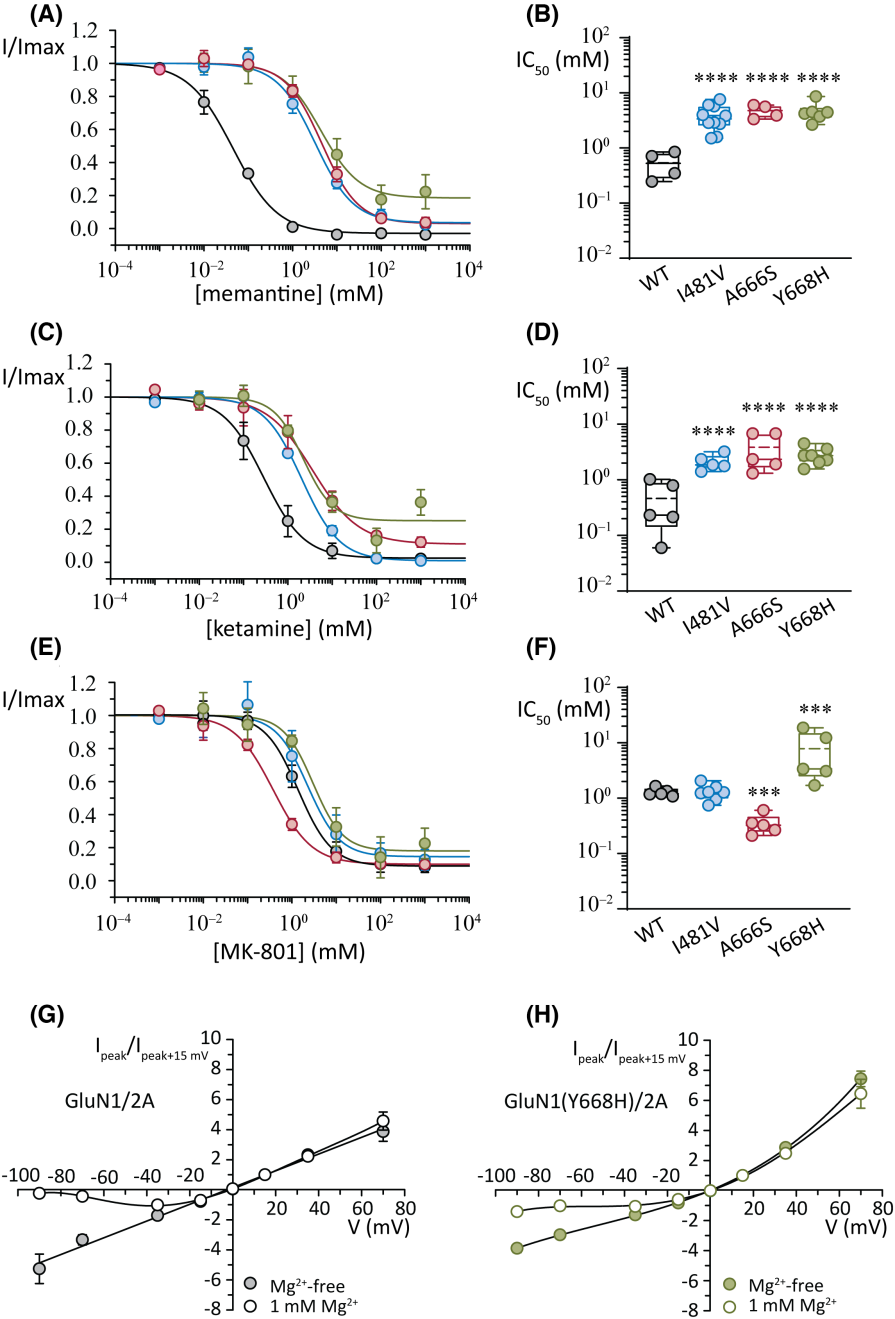
Effects of open channel blockers. (A, B) Concentration–response plots for peak current (A) and summary of the half‐maximal inhibitory concentration (IC_50_) values (B) for the inhibition by memantine. (C, D) Concentration–response plots for peak current (C) and summary of the IC_50_ values (D) for the inhibition by ketamine. (E, F) Concentration–response plots for peak current (E) and summary of the IC_50_ values (F) for the inhibition by MK‐801. Currents were obtained at a holding potential of −100 mV. (G) Normalized whole‐cell current–voltage plots for wild‐type (WT) GluN1/2A receptors in the absence (filled circles) and presence (open circles) of extracellular Mg^2+^ ions (1 mmol·L^−1^). Note the block of current at negative voltages in 1 mmol·L^−1^ Mg^2+^. (H) Normalized whole‐cell current–voltage plots for GluN1(Y668H)/2A variant receptors in the absence (filled circles) and presence (open circles) of extracellular Mg^2+^ ions (1 mmol·L^−1^). Note the impaired block at negative voltages in 1 mmol·L^−1^ Mg^2+^ and the greater relative outward current compared to WT receptors. Currents were normalized to +15 mV. ***p < .001, ****p < .0001.

Single‐receptor currents were recorded from outside‐out membrane patches excised from HEK293AD cells expressing WT, GluN1(A666S)/2A, or GluN1(Y668H)/2A variant receptors. Receptor activity was elicited by continuous application of a saturating concentration of agonists consisting of 1 mmol·L^−1^ glutamate plus 100 μmol·L^−1^ glycine (Figure 5A–C). WT receptors activated in bursts of activity with a unitary current amplitude of 3.3 ± .2 pA (*n* = 7 patches; Figure 5A). Similarly, the GluN1(A666S)/2A receptors opened to an amplitude of 3.4 ± .2 pA (*n* = 3 patches; Figure 5B). By contrast, the GluN1(Y668H)/2A variant receptors opened in relatively shorter bursts and to multiple levels within each burst (Figure 5C). These intra‐activation transitions in amplitude consisted of steps to small (.8 ± .1 pA), medium (2.5 ± .1 pA), and large (3.3 ± .1 pA) levels (*n* = 8 patches; Figure 5D). The small, medium, and large levels were significantly different from each other (ANOVA, *p* < .001 for each comparison), whereas the large level was not different from the main level in WT receptors (*p* = .628). At a reversal potential of 0 mV (see Figure 6G,H) and a liquid junction potential of 3.9 mV, we calculated the main conductance of the WT and GluN1(A666S)/2A receptors to be 45 pS and 46 pS, respectively, and for the GluN1(Y668H)/2A receptors to be 11 pS, 34 pS, and 45 pS.

We analyzed the durations of discrete receptor active periods and the proportion of time spent in conducting configurations during active periods (Open probability, *P*_O_) for the WT and the two pore variant receptors. WT receptors activated for a mean of 652 ± 845 ms (nine patches, 450 separate activations; Figure 5E) and a *P*_O_ of .67 ± .21 (Figure 5F), which are similar to previously determined values.^16^ GluN1(A666S)/2A receptors showed no change in these parameters relative to WT receptors, with an estimated mean active duration of 800 ± 1078 ms (three patches, 48 separate activations) and a *P*_O_ of .66 ± .23 (Figure 5E,F). By contrast, both of these single‐channel parameters were altered in the GluN1(Y668H) variant. The mean active duration of GluN1(Y668H)/2A receptors was 60 ± 52 ms (six patches, 1245 separate activations, *p* < .0001; Figure 5E), and the *P*_O_ was .49 ± .25 (*p* < .0001; Figure 5F).

### 3.7 Open channel block by memantine, ketamine, and MK‐801

As additional functional probes and to determine whether target site therapeutic drugs might be effective at correcting the functional deficits of the variants, we examined the potency of the clinically relevant pore blockers, memantine, ketamine, and MK‐801. Receptor activation was achieved by applying 35 μmol·L^−1^ glutamate and 10 μmol·L^−1^ glycine at each receptor.

Memantine blocked WT receptor current with a pIC_50_ of .32 ± .25 (IC_50_ of .54 ± .29 μmol·L^−1^) and an *n*_H_ of −1.3 ± .9 (*n* = 4; Figure 6A,B). At the GluN1(I481V)/2A variant, memantine blocked current with a reduced potency compared to WT receptors with a pIC_50_ (negative logarithm of IC_50_) of −.53 ± .24 (IC_50_ of 3.9 ± 2.0 μmol·L^−1^) and an *n*_H_ of −1.1 ± .3 (*n* = 10, ANOVA, *p* < .001). The two pore variants were also less sensitive to memantine block. The GluN1(A666S)/2A receptors had a pIC_50_ for memantine of −.66 ± .12 (IC_50_ of 4.7 ± 1.3 μmol·L^−1^) and an *n*_H_ of −1.1 ± .1 (*n* = 4, ANOVA, *p* < .001), and the GluN1(Y668H)/2A receptors had a pIC_50_ of −.64 ± .17 (IC_50_ of 4.7 ± 2.0 μmol·L^−1^) and an *n*_H_ of −.8 ± .2 (*n* = 6, ANOVA, *p* < .001).

Ketamine application at WT receptors blocked current with a pIC_50_ of .52 ± .50 (IC_50_ of .46 ± .42 μmol·L^−1^) and an *n*_H_ of −1.3 ± .6 (*n* = 5; Figure 6C,D). The potency with which ketamine blocked all three variants was significantly reduced compared to WT receptors (ANOVA, *p* < .001 for all variants). The GluN1(I481V)/2A variant receptors had an pIC_50_ of −.31 ± .14 (IC_50_ of 2.1 ± .7 μmol·L^−1^) and an *n*_H_ of −1.1 ± .4 (*n* = 5). The GluN1(A666S)/2A receptors were blocked by ketamine with a pIC_50_ of −.49 ± .33 (IC_50_ of 3.8 ± 2.7) and an *n*_H_ of −.95 ± .63 (*n* = 5), and the GluN1(Y668H)/2A receptors were blocked with a pIC_50_ of −.42 ± .15 (IC_50_ of 2.8 ± 1.0 μmol·L^−1^) and a Hill slope of −1.1 ± .2 (*n* = 7).

At WT receptors, MK‐801 had a pIC_50_ of −.20 ± .25 (IC_50_ of 1.9 ± 1.5 μmol·L^−1^) and an *n*_H_ of −1.2 ± .4 (*n* = 6) and the GluN1(I481V)/2A variant had a pIC_50_ and Hill slope of −.02 ± .25 (ANOVA, *p* = .946, IC_50_ of 1.2 ± .5 μmol·L^−1^) and −1.4 ± .6 (*n* = 8). However, the pore variants did change the potency of MK‐801. At GluN1(A666S)/2A receptors, MK‐801 had a pIC_50_ of .48 ± .17 (ANOVA, *p* = .002, IC_50_ of .35 ± .15 μmol·L^−1^) and an *n*_H_ of −.89 ± .34 (*n* = 5), suggesting an increase in potency of this blocker compared to WT receptors. Conversely, MK‐801 was less potent at the GluN1(Y668H)/2A receptors, with a pIC_50_ of −.72 ± .44 (ANOVA, *p* = .002, IC_50_ of 7.8 ± 7.4 μmol·L^−1^) and a Hill slope of −.95 ± .62 (*n* = 5). A summary of the group data for the concentration–response experiments and the IC_50_ comparisons for MK‐801 is shown in Figure 6E,F, respectively.

### 3.8 GluN1(Y668H)/2A reduces block by Mg^2+^ ions

The Y668H mutation is positioned near the apex of a conically shaped vestibule within the ion pore. The base of this vestibule is formed by the M2 domains, which contain critical determinants of block and permeation of Mg^2+^ and Ca^2+^ ions.^5^, ^19^, ^20^ We wished to investigate whether the substitution to histidine affected Mg^2+^ block at GluN1(Y668H)/2A receptors. Whole‐cell recordings were carried out over a range of voltages for WT and GluN1(Y668H)/2A variant receptors in the presence and absence of 1 mmol·L^−1^ extracellular Mg^2+^ ions for each recorded cell. WT GluN1/2A receptors in the absence of extracellular Mg^2+^ produced a linear current–voltage (I–V) relationship (Figure 6G). Substituting the Mg^2+^‐free extracellular with one containing 1 mmol·L^−1^ Mg^2+^ produced the expected voltage‐dependent current block at negative membrane voltages.

The I–Vs of the GluN1(Y668H)/2A variant receptors showed features that were distinct from those of the WT. The I–V in the presence of extracellular Mg^2+^ was outwardly rectifying. The outward currents were relatively larger by approximately 1.9‐fold (Figure 6H) compared to the I–Vs of WT receptors and approximately fivefold greater relative to the I–Vs in the absence of Mg^2+^. The GluN1(Y668H)/2A variant also showed impaired Mg^2+^ block at negative membrane voltages in the presence of 1 mmol·L^−1^ Mg^2+^. The enhanced outward currents and current‐carrying capacity at negative voltages are gain‐of‐function properties of this variant.

## 4 DISCUSSION

Clinical features of the three patients harboring the *GRIN1* gene variants described in this study include severe intellectual disability, developmental delay, and hypotonia. Notably, other patients expressing missense variants to the GluN1(A666) and GluN1(Y668) positions exhibit similar clinical features to those described here.^10^, ^12^ The p.Ala666Ser and p.Tyr668Ser variants were recently studied in heterologous cells on the GluN1‐4a background (numbered as p.Ala645Ser and p.Tyr647Ser). When expressed as GluN1‐4a(A645S)/2A receptors, glycine and glutamate sensitivity remained unaffected, as did surface expression.^15^ Our functional analysis shows a small (~3‐fold) but significant increase in sensitivity to glycine when the same variant is expressed in the GluN1‐3b splice isoform along with the GluN2A subunit. The increase in glycine sensitivity is a gain‐of‐function phenotype and likely to enhance excitatory input, particularly from extrasynaptic receptors that mediate tonic excitation. The GluN1‐4a(Y647S)/2A receptors failed to reach the cell membrane^15^ and on that basis were classified as a loss‐of‐function phenotype.^12^ The GluN1‐3b(Y668H)/2A receptors showed reduced levels of expression at the cell membrane, consistent with impaired receptor trafficking, but as it was only partial, it did not preclude functional analysis of receptors. The substitution to a histidine (rather that a serine) resulted in GluN1(Y668H)/2A receptors exhibiting an increase in glutamate and glycine sensitivities by an order of magnitude.

We observed that the concentration–response relationship for glycine and glutamate for the GluN1(Y668H)/2A variant did not diminish to zero as was observed for the other receptors. Follow‐up experiments showed that this variant was activated by application of either glutamate or glycine without added coagonist. We infer that either coagonists were present in our stocks but at levels that were below the detection threshold of our mass spectrometer or, more remarkably, that the GluN1(Y668H)/2A receptor can be activated by either neurotransmitter alone. This would enhance both tonic (extrasynaptic) and phasic (synaptic) excitatory input during development.

Our single‐receptor recordings revealed two altered functional properties of the GluN1(Y668H)/2A variant receptors that are compatible with GluN1(Y668) being a hub that couples receptor activation and ionic conductance. First, discrete active periods of individual receptors were relatively brief and occurred as frequent “chatter” throughout the recordings, which is consistent with enhanced receptor gating and the observed increase in sensitivity to neurotransmitters. Second, single‐receptor active periods were characterized by multiple transitions in amplitude, suggesting that the GluN1(Y668) position also functions as a site where single‐channel conductance is regulated.

Open channel blockers of NMDA receptors, including memantine, ketamine, and MK‐801, share an overlapping, conically shaped binding pocket. Ketamine forms hydrophobic interactions with GluN1‐V665 and A666, whereas memantine interacts with GluN1‐V665 and T669^21^ and MK‐801 interacts principally with GluN1‐V665.^22^ These differences in molecular interactions are subtle and reflect the similar decrease in potency for the M3 pore variants. A decrease in potency of memantine and ketamine was observed for the GluN1(I481V)/2A receptors, even though neither blocker interacts directly with the GluN1‐481 position. These data indicate a retrograde functional coupling between the ion channel gate and the extracellular domain around the GluN1‐481 position, as has been observed for open channel block by picrotoxin at anion‐selective γ‐aminobutyric acid type A (GABA_A_) receptors, which is accompanied by structural changes in the extracellular domain of the receptor.^23^

Our study provides new insights into molecular pathogenic mechanisms of three GluN1 subunit variants associated with developmental delay and epilepsy and has implications for treatment options for the affected individuals. All three individuals have been receiving therapies that either potentiate GABA_A_ receptor‐mediated inhibitory input or reduce neuronal firing. All three individuals have shown limited benefit from current treatments. Our data show that clinically relevant NMDA receptor blockers might be more efficacious at alleviating symptoms even though the potency and efficacy of the blockers has been compromised by the variant receptors.

## AUTHOR CONTRIBUTIONS

Angelo Keramidas, Lotten Ragnarsson, and Victor Anggono contributed to the conception and design of the study. Angelo Keramidas, Lotten Ragnarsson, Zihan Zhang, Sooraj S. Das, Poanna Tran, and Åsa Andersson contributed to the acquisition and analysis of data. Vincent des Portes, Cecilia Desmettre Altuzarra, Ganaelle Remerand, Audrey Labalme, Nicolas Chatron, Damien Sanlaville, and Gaetan Lesca contributed patient genetic screening and clinical information. Angelo Keramidas, Lotten Ragnarsson, Victor Anggono, and Irina Vetter contributed to drafting a significant portion of the manuscript or figures.

## FUNDING INFORMATION

This work was supported by grants from the National Health and Medical Research Council (GNT1156673 to A.K.), the Australian Research Council (ARC; DP190101390 to V.A. and A.K.), and the Australian Medical Research Future Fund (Clem Jones Centre for Aging Dementia Research Flagship Project Grant to V.A.). V.A. is supported by an ARC Future Fellowship (FT220100485). S.S.D. is supported by a Research Training Program Scholarship from the Australian Government and the University of Queensland.

## CONFLICT OF INTEREST STATEMENT

The authors report no competing interests. We confirm that we have read the Journal's position on issues involved in ethical publication and affirm that this report is consistent with those guidelines.

## Supporting information


FIGURE S1

DATA S1

## References

[1] Wang H , Lv S , Stroebel D , Zhang J , Pan Y , Huang X , et al. Gating mechanism and a modulatory niche of human GluN1–GluN2A NMDA receptors. Neuron. 2021;109:2443–2456.e2445. 10.1016/j.neuron.2021.05.031 34186027

[2] Paoletti P , Bellone C , Zhou Q . NMDA receptor subunit diversity: impact on receptor properties, synaptic plasticity and disease. Nat Rev Neurosci. 2013;14:383–400. 10.1038/nrn3504 23686171

[3] Traynelis SF , Wollmuth LP , McBain CJ , Menniti FS , Vance KM , Ogden KK , et al. Glutamate receptor ion channels: structure, regulation, and function. Pharmacol Rev. 2010;62:405–496. 10.1124/pr.109.002451 20716669 PMC2964903

[4] Ascher P , Nowak L . The role of divalent cations in the *N*‐methyl‐d‐aspartate responses of mouse central neurones in culture. J Physiol. 1988;399:247–266. 2457089 10.1113/jphysiol.1988.sp017078 PMC1191662

[5] Burnashev N , Schoepfer R , Monyer H , Ruppersberg JP , Günther W , Seeburg PH , et al. Control by asparagine residues of calcium permeability and magnesium blockade in the NMDA receptor. Science. 1992;257:1415–1419. 1382314 10.1126/science.1382314

[6] Bossi S , Dhanasobhon D , Ellis‐Davies GCR , Frontera J , de Brito van Velze M , Lourenço J , et al. GluN3A excitatory glycine receptors control adult cortical and amygdalar circuits. Neuron. 2022;110:2438–2454.e2438. 10.1016/j.neuron.2022.05.016 35700736 PMC9365314

[7] Hansen KB , Yi F , Perszyk RE , Furukawa H , Wollmuth LP , Gibb AJ , et al. Structure, function, and allosteric modulation of NMDA receptors. J Gen Physiol. 2018;150:1081–1105. 10.1085/jgp.201812032 30037851 PMC6080888

[8] Amin JB , Moody GR , Wollmuth LP . From bedside‐to‐bench: what disease‐associated variants are teaching us about the NMDA receptor. J Physiol. 2020;599:397–416. 10.1113/JP278705 32144935 PMC7483363

[9] Camp CR , Yuan H . *GRIN2D*/GluN2D NMDA receptor: unique features and its contribution to pediatric developmental and epileptic encephalopathy. Eur J Paediatr Neurol. 2020;24:89–99. 10.1016/j.ejpn.2019.12.007 31918992 PMC7035963

[10] Garcia‐Recio A , Santos‐Gómez A , Soto D , Julia‐Palacios N , García‐Cazorla À , Altafaj X , et al. *GRIN* database: a unified and manually curated repertoire of *GRIN* variants. Hum Mutat. 2021;42:8–18. 10.1002/humu.24141 33252190

[11] Brock S , Laquerriere A , Marguet F , Myers SJ , Hongjie Y , Baralle D , et al. Overlapping cortical malformations in patients with pathogenic variants in *GRIN1* and *GRIN2B* . J Med Genet. 2022;60:183–192. 10.1136/jmedgenet-2021-107971 35393335 PMC10642159

[12] Lemke JR , Geider K , Helbig KL , Heyne HO , Schütz H , Hentschel J , et al. Delineating the *GRIN1* phenotypic spectrum: a distinct genetic NMDA receptor encephalopathy. Neurology. 2016;86:2171–2178. 10.1212/WNL.0000000000002740 27164704 PMC4898312

[13] Jones KS , VanDongen HM , VanDongen AM . The NMDA receptor M3 segment is a conserved transduction element coupling ligand binding to channel opening. J Neurosci. 2002;22:2044–2053. 11896144 10.1523/JNEUROSCI.22-06-02044.2002 PMC6758261

[14] Yuan H , Erreger K , Dravid SM , Traynelis SF . Conserved structural and functional control of *N*‐methyl‐d‐aspartate receptor gating by transmembrane domain M3. J Biol Chem. 2005;280:29708–29716. 10.1074/jbc.M414215200 15970596

[15] Kolcheva M , Kortus S , Krausova BH , Barackova P , Misiachna A , Danacikova S , et al. Specific pathogenic mutations in the M3 domain of the GluN1 subunit regulate the surface delivery and pharmacological sensitivity of NMDA receptors. Neuropharmacology. 2021;189:108528. 10.1016/j.neuropharm.2021.108528 33773999

[16] Yong XLH , Zhang L , Yang L , Chen X , Tan JZA , Yu X , et al. Regulation of NMDA receptor trafficking and gating by activity‐dependent CaMKIIalpha phosphorylation of the GluN2A subunit. Cell Rep. 2021;36:109338. 10.1016/j.celrep.2021.109338 34233182 PMC8313361

[17] Anggono V , Clem RL , Huganir RL . PICK1 loss of function occludes homeostatic synaptic scaling. J Neurosci. 2011;31:2188–2196. 10.1523/JNEUROSCI.5633-10.2011 21307255 PMC3071039

[18] Kolcheva M , Ladislav M , Netolicky J , Kortus S , Rehakova K , Krausova BH , et al. The pathogenic N650K variant in the GluN1 subunit regulates the trafficking, conductance, and pharmacological properties of NMDA receptors. Neuropharmacology. 2022;222:109297. 10.1016/j.neuropharm.2022.109297 36341805

[19] Wollmuth LP , Kuner T , Sakmann B . Adjacent asparagines in the NR2‐subunit of the NMDA receptor channel control the voltage‐dependent block by extracellular Mg^2+^ . J Physiol. 1998;506(Pt 1):13–32. 9481670 10.1111/j.1469-7793.1998.013bx.x PMC2230696

[20] Premkumar LS , Auerbach A . Identification of a high affinity divalent cation binding site near the entrance of the NMDA receptor channel. Neuron. 1996;16:869–880. 8608005 10.1016/s0896-6273(00)80107-5

[21] Chou TH , Epstein M , Michalski K , Fine E , Biggin PC , Furukawa H . Structural insights into binding of therapeutic channel blockers in NMDA receptors. Nat Struct Mol Biol. 2022;29:507–518. 10.1038/s41594-022-00772-0 35637422 PMC10075384

[22] Song X , Jensen MØ , Jogini V , Stein RA , Lee CH , Mchaourab HS , et al. Mechanism of NMDA receptor channel block by MK‐801 and memantine. Nature. 2018;556:515–519. 10.1038/s41586-018-0039-9 29670280 PMC5962351

[23] Masiulis S , Desai R , Uchański T , Serna Martin I , Laverty D , Karia D , et al. GABAA receptor signalling mechanisms revealed by structural pharmacology. Nature. 2019;565:454–459. 10.1038/s41586-018-0832-5 30602790 PMC6370056

