# Supplementary material for: GRIN1 variants associated with neurodevelopmental disorders reveal channel gating pathomechanisms
Source: Epilepsia. 2023 Oct 17;64(12):3377–88. doi: 10.1111/epi.17776 (PMC10952597; doi:10.1111/epi.17776)
Supplement: Supplementary file 1 — FIGURE S1 [file EPI-64-3377-s002.docx]

**Supporting Figure 1**


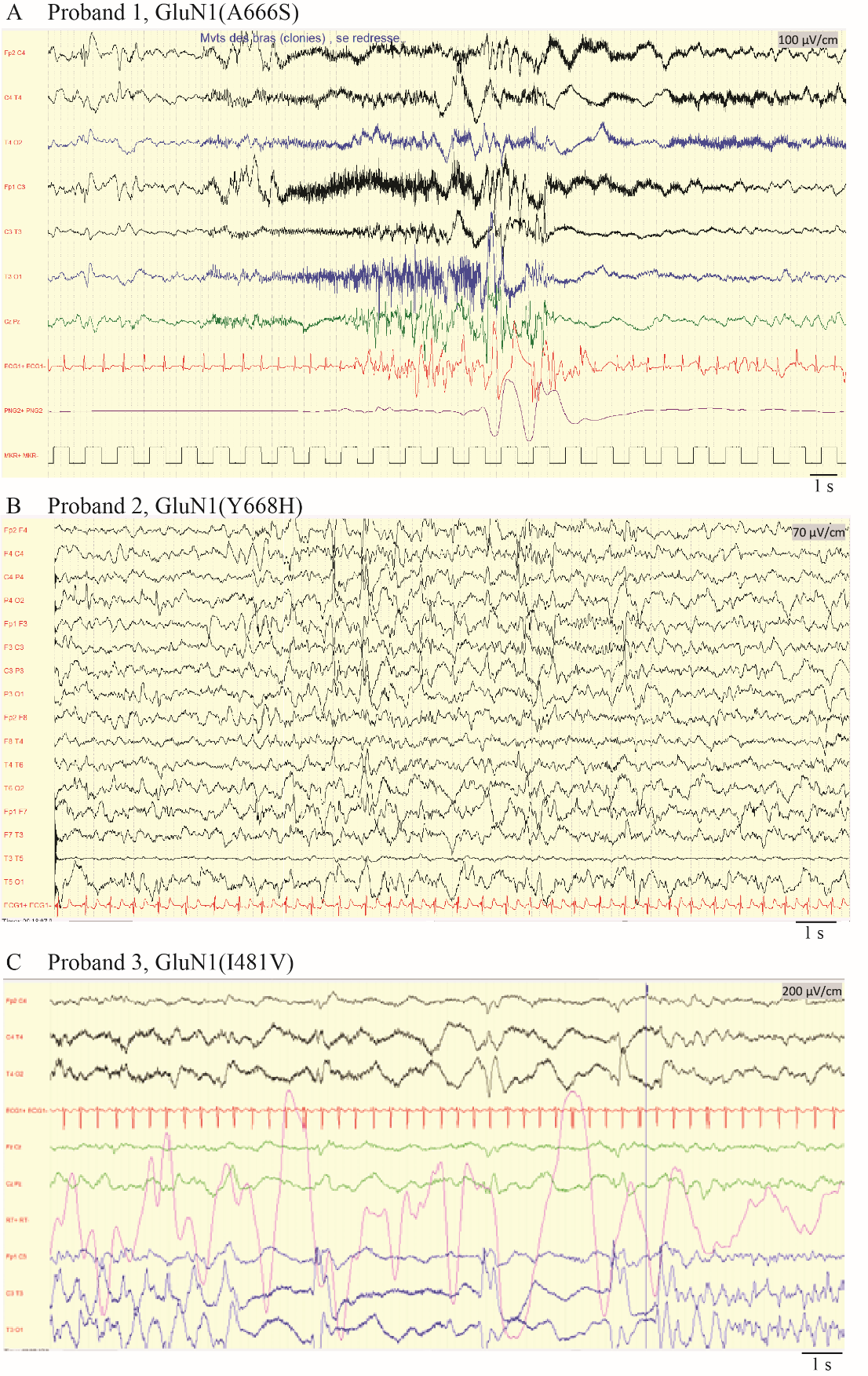


**Supporting Figure 1. Proband EEGs.** (**A**) EEG of proband 1 at 15 years old showing generalised tonic-clonic seizure activity. (**B**) EEG of proband 2 at 20 months of age when the left temporal arachnoid cyst was detected. (**C**) EEG of proband 3 showing left temporal seizures during an episode of status epilepticus at 22 months of age.
