# Supplementary material for: GRIN1 variants associated with neurodevelopmental disorders reveal channel gating pathomechanisms
Source: Epilepsia. 2023 Oct 17;64(12):3377–88. doi: 10.1111/epi.17776 (PMC10952597; doi:10.1111/epi.17776)
Supplement: Supplementary file 2 — DATA S1 [file EPI-64-3377-s001.docx]

Supporting information

# Clinical presentation of patients

**Proband 1** (female)**:** Genetic screening identified a *de novo* missense variant in the *GRIN1* gene (c.1996G>T, p.Ala666Ser). The proband expressing the GluN1(A666S) variant has severe intellectual disability, motor delay, cortical visual impairment and no language. Beginning at age 2, the proband experienced focal seizures with fronto-central onset, tonic-clonic seizures or seizures involving loss of eye contact, accompanied by chewing motions. At age 6, the proband experienced only nocturnal seizures and responded to levetiracetam, which reduced seizure incidence up until the age of 10, after which seizures became daily and were characterised by hypertonic upper limb postures.

Topiramate and vigabatrin had no effect on seizures, whereas upon administration of lamotrigine and perampanel, the patient experienced seizure aggravation. The current treatment regimen includes levetiracetam, clobazam, diphenylhydantoine (Phenytoin) and cannabidiol. The proband continues to have 3-6 tonic-clonic seizures per day (supporting Figure 1A) with associated hypertonia in the lower limbs.

## Proband 2 (female): Genetic testing revealed a *de novo* missense variant to the *GRIN1* gene (c.2002T>C, p.Tyr668His). This proband has developmental and language delay. An MRI scan at 20 months revealed a left temporal arachnoid cyst (supporting Figure 1B) that required surgical intervention. At four years of age the proband developed secondary aphasia. Generalised tonic or tonic-clonic seizures first developed at the age of nine. The proband continues to have approximately one seizure per month that can be either generalised tonic-clonic or involve episodes of staring.

## Levetiracetam treatment was ineffective and the proband continues to be treated with carbamazepine and perampanel with only partial reduction in seizure incidence.

**Proband 3** (female): This proband has global developmental and language delay as well as psychomotor agitation, aggressivity and food selectivity. A missense variant to the *GRIN1* gene (c.1441A>G, p.Ile481Val) was identified from a genetic screen. The first seizure occurred at 13 months, followed by another involving eye clonia and hypotonia at 18 months. Status epilepticus occurred at 20 months (right hemispheric), 22 months (left temporal, supporting Figure 1C), 24 months (generalised) and at 37 months, following the withdrawal of clobazam. Clobazam was resumed after the last episode of status epilepticus.

The proband was given valproate at 13 months. Clonazepam was effective in relieving the first episode of status epilepticus. Clobazam was added to valproate at 20 months. After the second episode of status epilepticus levetiracetam was included with clobazam and valproate. At 24 months the dosage of levetiracetam was increased. The proband continues to experience generalised seizures.

**Statistical Analysis**

ANOVA analyses were used to compare data from multiple groups. A one-way ANOVA was used for normally distributed data (unitary currents), whereas a Kruskal-Wallis one-way ANOVA was used for exponentially distributed data (active durations and P_O_s). Unpaired *t*-tests were used to compare Hill fit parameters.
